# Anthropometric Measurements of Foot in Undergraduate Medical Students of a Medical College: A Descriptive Cross-sectional Study

**DOI:** 10.31729/jnma.8701

**Published:** 2024-08-31

**Authors:** Niraj Pandey, Deepak Chaudhary, Sanjay Kumar Yadav

**Affiliations:** 1 Department of Anatomy, Devdaha Medical College and Research Institute, Bhaluhi, Rupandehi, Nepal

**Keywords:** *anthropometry*, *foot breadth*, *foot index*, *foot length*, *Nepal*

## Abstract

**Introduction:**

Anthropometry is one of the important parameters for differentiation of sex which varies significantly based on hereditary, geographical, racial, sexual, and other factors. This study was done to provide baseline foot anthropometric data for Nepali medical students to improve the accuracy of stature estimations and enhance forensic and clinical applications. The aim of the study was to calculate anthropometric measurements of foot in undergraduate medical students of a medical college.

**Methods:**

A descriptive cross-sectional study was conducted on medical students studying at a tertiary care hospital during the period of November 6, 2022 to February 28, 2023 after obtaining ethical clearance from the Institutional Review Committee (Reference number: 06/2022). A total population sampling method was used. The present study was conducted to determine the mean foot index from the right and left foot, mean measurements of the left and right foot, and sex-related dimensions of the foot in Nepalese medical students. Data were collected and analyzed using SPSS.

**Results:**

Out of 115 participants, the mean foot index for male and female were 38.34±2.13 and 39.33±2.22 respectively. The mean length of the right foot for males and females were 24.12±0.98 cm and 22.10±1.25 cm respectively. The mean length of the left foot for males and females was 24.20±1.06 cm and 22.07±1.24 cm respectively.

**Conclusions:**

This study provides mean values of different measurements of the right and left foot of both sexes of the age group of 18-24 years in the students of a medical college.

## INTRODUCTION

Humans are unique among primates for their upright posture and bipedal locomotion, with stature estimation traditionally relying on measurements of long bones, hand, and foot.^1^ Key to human identification, anthropometric measurements—including age, sex, stature, and race—are central to forensic anthropology and personal identification.^1^

The clinical importance of the foot is indicated by the emergence of "podiatry" which deals with the study and care of the foot.^2^ Anthropometric data vary considerably for individuals, within a family or a nation, and in between nations.^3^ Foot measurements have proven to be more accurate than long bones for predicting height, highlighting their importance in anthropometric and forensic analysis.^4^ Despite their potential, baseline foot anthropometric data specific to Nepali populations, particularly medical students, is lacking.

This study aimed to study foot anthropometric data of medical students a medical college Nepal.

## METHODS

A descriptive cross-sectional study was carried from November 6, 2022 to February 28, 2023 in the Department of Anatomy out after approval from Institutional Review Committee of Devdaha Medical College and Research Institute (DMCRI) (Reference number: 06/2022). Medical students enrolled in the institute within age group 18 to 24 years were included whereas those with malformations, deformities, trauma, or any other abnormalities were excluded from the study. Total sampling was done and a total number of 115 medical students were taken. Informed consent was taken from each participant.

Standing height was measured by a stadiometer against the wall barefoot, with their heels together, buttocks and back touching the stadiometer. Foot length was measured by a foot caliber. It was measured when the subject was sitting in a relaxed position putting the same weight on both feet after taking off the shoes, socks, and stockings. The fixed jaw of the caliber was placed at pternion and the sliding jaw was fixed at acropodion. The caliber was kept parallel to the long axis of the foot. Foot width or breadth was measured by maintaining the same posture and condition by the foot caliber. The sliding and fixed jaws of the caliber were placed on the metatarsal tibiale and metatarsal fibulare, respectively. The caliber was kept perpendicular to the long axis of the foot. The sliding and fixed jaws of the caliber were placed on the medial and lateral surfaces of the heel from behind to measure the heel breadth, respectively. The circumference of the foot was measured at the ball of the foot which corresponds with metatarsal tibiale and metatarsal fibulare of the foot at a perpendicular plane to the long axis of the foot using the flexible measuring tape. All measurements were performed thrice and the mean value was obtained. A fixed time 11 AM to 4 PM was selected for the physical measurement to eliminate the discrepancies due to diurnal variation. The foot index was calculated by using a formula. Foot index=foot breadth divided by foot length and multiplied by a hundred.^5^ Bony landmarks acropodian, metatarsal tibiale, instep, sphyrion, pternion, and metatarsal fibulare are marked in each subject for measurements.^6^ After measurements, the data was collected using a prestructured proforma.

The collected data was tabulated, and statistically analyzed by using SPSS. The minimum, maximum, mean and standard deviations were calculated.

## RESULTS

A total of 115 students of Devdaha Medical College and Research Institute were studied where 80 (69.56%) were male and 35 (30.43%) were female. The mean age of the students recruited were 19.97±1.02 years ranging from 18 to 23 years of age. The mean of foot length, foot width, ball circumference and foot heel were 23.51 ±1.41 cm, 9.09±0.62 cm, 24.11 ±1.69 cm, 5.80±0.50 cm on the right side, and 23.55±1.45 cm, 9.06±0.59 cm, 23.81±1.67 cm, 5.79±0.47 cm on the left side respectively (Table 1).

**Table 1:** Anthropoment of medical students (n = 115).

Variables	Minimum	Maximum	Mean	Standard deviation
Weight of students (kg)	39	86	56.09	9.37
Height of students(cm)	141	182.50	163.04	8.58
Right foot length(cm)	18.60	26.70	23.51	1.41
Left foot length(cm)	18.60	27	23.55	1.45
Right-foot width (cm)	7.50	11	9.09	0.62
Left-foot width (cm)	7.50	10.50	9.06	0.59
Right-ball circumference (cm)	20	28.10	24.11	1.69
Left-ball-circumference (cm)	19.50	27.50	23.81	1.67
Right-foot heel (cm)	4.40	6.80	5.80	0.50
Left-foot heel (cm)	4.60	6.80	5.79	0.47

The mean of foot length, foot width, ball circumference and foot heel in male students were 24.12±0.98 cm, 9.28±0.54 cm, 27.55±1.29 cm, 6.05±0.41 cm on the right side and 24.20±1.00 cm, 9.23±0.53 cm, 24.58±1.17 cm, 5.98±0.41 cm on the left side, respectively (Table 2).

**Table 2:** Anthropometric measurements for male medical students (n = 80).

Variables in male	Minimum	Maximum	Mean±SD
Height of students (cm)	149.50	182.50	167.17±6.03
Body weight (kg)	45	86	59.61±8.05
Right foot length (cm)	21.10	26.70	24.12±0.98
Right foot width (cm)	8	11	9.28±0.54
Right foot heel (cm)	5.10	6.80	6.05±0.41
Right ball circumference (cm)	21.30	28.10	27.55±1.29
Left foot length (cm)	21.20	27	24.20±1.00
Left foot width (cm)	7.80	10.50	9.23±0.53
Left foot heel (cm)	4.90	6.80	5.98±0.41
Left ball circumference (cm)	21.20	27.50	24.58±1.17

The mean of foot length, foot width, ball circumference, and foot heel in female students were 22.10±1.25 cm, 8.67±0.59 cm, 22.40±1.18 cm, 5.34±0.37 cm on the right side and 22.07±1.24 cm, 8.68±0.56 cm, 22.04±1.23 cm, 5.37±0.33 cm on the left side, respectively (Table 3).

**Table 3:** Anthropometric measurements for measurements for female medical students (n = 35).

Variable in female	Minimum	Maximum	Mean±SD
Height of students (cm)	141	163	153.61±5.51
Body weight (kg)	39	72	48.05±6.96
Right foot length (cm)	18.60	24.90	22.10±1.25
Right foot width (cm)	7.50	9.60	8.67±0.59
Right foot heel (cm)	4.40	6.20	5.34±0.37
Right ball circumference (cm)	20	24.50	22.40±1.18
Left foot length (cm)	18.60	24.70	22.07±1.24
Left foot width (cm)	7.50	9.80	8.68±0.56
Left foot heel (cm)	4.60	6.10	5.37±0.33
Left ball circumference (cm)	19.50	24.40	22.04±1.23

The mean foot index was 38.34±2.13 and 39.33±2.22, for males and females respectively and the mean foot index of all students was 38.64±2.20.

## DISCUSSION

Determining stature from incomplete and decomposing skeletal remains plays a vital role in personal identification. This study was carried out to provide anthropometric data for foot length and foot breadth of young adult Nepalese to add to existing data. The human foot must be stable to support body weight while standing, flexible for walking, and adaptable to variations in surface. The data from our study could be valuable for anthropological research, forensic science, genetic studies, and clinical medical practices, such as reconstructive surgery. The estimation of height from various long bones, head length, and hand length has been attempted by many workers. However, foot dimensions have not frequently been used for this. Body morphology and height changes are influenced by many internal and external factors, like mechanical effects of use and wear and physical stress during the life of a person, different cultures, and lifestyles.^7^ The knowledge of normal measurements of foot length and foot breadth may be useful for orthopedic surgeons, forensic purposes, and for the industrial manufacturing of foot prostheses.

It is helpful in forecasting the height from the foot length and foot breadth when only these body parts are available. The normal human foot shows great individual variation in length, and breadth in males and females.^8^ The study conducted in Thailand, West Bengal, and North India shows that body stature, body weight, foot length, and foot width of the adult male were near about similar to our present study.^9-11^ In other studies, the mean stature, body weight, and foot length of males were higher and the mean foot width was similar to that of the present study.^12,13^

The mean stature body weight, foot width, and foot length of Slovakian were higher than in our present study.^14^ The mean foot index of our study was 38.34±2.13 for male and 39.33±2.22 for female. In another study conducted on the Nepalese population, the mean foot index for males and females was 39.24±3.78 and 39.89±4.82 respectively.^15^ The same study showed a strong positive correlation between height and foot length.^15^ The Hong Kong Chinese male had higher mean stature, and mean foot length but similar foot width in comparison to our study.^16,17^ In our study, the male and female foot dimensions are comparatively lower than the study done in Nigerian populations.^18,19^

A study conducted in Nepal reported the mean value for right foot length to be 23.25±1.89 cm and left foot length to be 23.23±1.88 cm. Similarly, the mean value for right foot breadth was found to be 9.19±1.07 cm, and left foot breadth was 9.20±1.07 cm, the mean value for foot index was 39.24±3.78 among male and 39.89±4.82 among female for both right and left feet.^20^ Our study showed similar findings which included the mean foot length and width to be 23.51±1.41 cm and 9.09±0.62 cm, respectively, on the right side, and 23.55±1.45 cm and 9.06±0.59 cm, respectively, on the left side, for both males and females. The mean foot index was found to be 38.34±2.13 for males and 39.33±2.22 for females, with an overall mean foot index of 38.64±2.20 for both sexes combined.

This study will provide a basis for comparison in future research on the Nepali population. This study is limited to medical students of one medical school with limited data set, therefore, the findings cannot be generalized. Broader inclusion consisting of various ethnic and regional population needs to be included for better generalization.

## CONCLUSIONS

The present anthropometric study was an attempt to construct data on different dimensions of the foot of the adult Nepalese population. Despite the limited sample size, this study offers a preliminary baseline for foot anthropometry in Nepalese adults aged 18 to 24. Future research with larger and more diverse samples is necessary to confirm these findings and further explore the factors influencing foot dimensions.
